# Hopelessness and HIV infection: an exploratory study with a gender-specific perspective

**DOI:** 10.1186/s40359-022-00755-2

**Published:** 2022-02-28

**Authors:** Lena Nilsson Schönnesson, Michael W. Ross, Diego Garcia-Huidobro, Lars E. Eriksson, Galit Andersson, Mark L. Williams, Anna-Mia Ekström

**Affiliations:** 1 Department of Global Public Health, Karolinska Institutet, 171 77 Stockholm, Sweden; 2 Joycelyn Elders Professor and Chair of Sexual Health Education, Department of Family Medicine, University of Minnesota Medical School, Minneapolis, MN USA; 3 Departamento de Medicina Familiar, Escuela de Medicina, Pontificia Universidad Catolica de Chile, Santiago, Chile; 4 Department of Neurobiology, Care Sciences and Society, Karolinska Institutet, SE-141 83 Huddinge, Sweden; 5 School of Health Sciences, City, University of London, London, EC1V 0HB UK; 6 Medical Unit Infectious Diseases, Karolinska University Hospital, 141 86 Stockholm, Sweden; 7 Joycelyn Elders Professor and Dean, Fay W. Boozman College of Public Health, University of Arkansas for Medical Sciences, Little Rock, AR USA; 8 Kolstan, Övre Kolstan, 671 98 Gunnarskog, Sweden

**Keywords:** Hopelessness, People living with HIV, Contributors, Gender differences, Sweden

## Abstract

**Background:**

An understudied psychological response to HIV-related stressors among people living with HIV is hopelessness. Hopelessness is the expectation that things will not improve and feeling helpless to change one’s current situation. The aim of this study was to assess prevalence and levels of hopelessness and its direct and indirect contributors in people living with HIV in Sweden.

**Methods:**

Participants included 967 women and men from the “Living with HIV in Sweden” cross-sectional study with available data regarding hopelessness measured by the Beck Hopelessness Scale. Binary and multiple logistic regression analyses were used to determine direct and indirect factors that may contribute to feelings of hopelessness. Path analyses were used to assess the underlying structure of hopelessness. All analyses were conducted by gender.

**Results:**

Almost half the participants reported moderate to severe hopelessness. There were no differences in frequency of feeling hopeless or level of hopelessness by gender or sexual orientation. Dissatisfaction with finances, dissatisfaction with physical health, and low HIV-related emotional support were found to be directly associated with hopelessness for both women and men. Although having some indirect factors in common, unemployment and HIV stigmatization, women and men had different underlying structures of hopelessness.

**Conclusions:**

Our findings are important to HIV clinicians in identifying those at risk of hopelessness from a gender perspective in order to reduce preventable psychological distress among people living with HIV.

## Background

The majority of people living with HIV in the West are on antiretroviral therapy (ART) achieving and maintaining undetectable viral loads and therefore have close to normal life expectancy and are not at risk of transmitting HIV to HIV-negative sex partners. Yet, many people living with HIV are confronted with HIV-related medical, psychosocial, sexual, and existential stressors. A high level of physical and psychosocial stressors can lead to a number of psychological outcomes, including feelings of hopelessness [1–3]. Numerous studies have examined the prevalence of and the factors associated with hopelessness in patients with various chronic diseases including cancer [4–6]. Yet, there has been relatively little research of hopelessness in people living with HIV.

According to the Diagnostic and Statistical Manual of Mental Disorders, 5th edition [DSM-5]; [7], hopelessness is a symptom of depressive disorders. However, hopelessness may occur independent of depression [4], or as a precursor to a subtype of depression called hopelessness depression [8]. Several studies have shown a person can have feelings of hopelessness without feeling depressed [4].

Hopelessness, triggered by intrapsychic crisis and/or external stressors [9], is indicated by expectations of negative outcomes and feelings of helplessness to change those expectations [8]. Beck et al. [10] described three dimensions of hopelessness: negative future expectations (the cognitive dimension), negative feelings about the future (the affective dimension), and negative thoughts and feelings about one’s ability to change or improve the future (the motivational dimension).

A number of factors have been found to be associated with feelings of hopelessness. Demographic characteristics include being female, single, or Afro-American. Low income or lack of HIV-related emotional support are associated, as is poor physical functioning, side-effects from ART, and poorer ART adherence. Hopelessness has also been found to be associated with internalized HIV stigmatization or psychological symptoms associated with a recent HIV diagnosis [11–16].

Hopelessness in people living with HIV is a significant area of study, as it can negatively affect sexual satisfaction [17], quality of life [15], and life satisfaction [14]. A Swedish study among people living with HIV found hopelessness was the principal predictor of quality of life [18]. The aim of the present study was to assess prevalence and levels of hopelessness and its direct and indirect contributors in people living with HIV in Sweden. Accumulated research evidence has recognized gender differences in the general population regarding psychosocial and life situation variables [19–21]. Thus, we decided to examine women and men separately as only statistically controlling for gender is unable to allow specific focus on such differences [22]. Most studies investigating hopelessness among people living with HIV have used exclusively male samples. The few studies that have included women and men in their samples did not examine gender specific correlates or had limited discussion about the impact of gender on feelings of hopelessness.

## Methods

### Participants and procedure

This study utilized data from a large nation-wide, cross-sectional study ‘‘Living with HIV infection in Sweden’’ examining quality of life and its correlates through an anonymous self-reported questionnaire executed between December 2013 and August 2014. The questionnaire including 77 items translated into ten languages. It was pilot tested in collaboration with Swedish non-governmental HIV organizations. All study procedures were approved by the Regional Ethical Board, Stockholm (DNR 2013/1552-31/4).

Participants were enrolled in the study from 15 infectious disease outpatient clinics across Sweden and two needle and syringe exchange clinics in Stockholm. These 17 clinics account for 75% of the HIV care provided in Sweden. Patients in the clinics were eligible to participate if they were 18 years old and had been diagnosed with HIV for at least six months.

All consecutive outpatients visiting their HIV unit were informed about the study and invited to participate in the study. Altogether, 1096 women and men completed the questionnaire. The response rate was about one quarter of these who could have attended the 17 HIV units at the time of data collection. No incentive was given. Recruitment and data collection procedures are described in further detail elsewhere [17, 18].

The present study analyzed data from 967 participants (89%) who had complete data on the Beck Hopelessness Scale.

### Measures

Hopelessness was measured using the Beck Hopelessness Scale [10]. The structure of the Beck’ Hopelessness Scale has been validated in Sweden on a clinical sample [23]. The scale is designed to measure the cognitive, affective, and motivational dimensions of hopelessness during the last seven days. The scale is a 20-item, self-administered questionnaire. All items are scored on a true–false rating scale. After recoding negatively worded items, the number of endorsed items is combined to a sum-score; the higher scores the greater hopelessness levels. The participants were classified into: no hopelessness (scores 0–3), mild hopelessness (scores 4–8), moderate hopelessness (scores 9–14) and severe hopelessness (scores 15–20) [10]. Cronbach’s α in this sample was very good, 0.91 (mean scale score 5.44, median 4.00, SD 5.14).

In addition to hopelessness, we asked participants to describe their sociodemographic characteristics— including gender, sexual orientation, relationship status, birth country, education, and employment status. Satisfaction with their financial situation was measured by a statement derived from the Life Satisfaction Scale [24]. We also measured clinical and psychosocial factors. Clinical factors included time since HIV diagnosis (0 to 10 years/more than 10 years), bothersome physical ART side-effects (yes/no), impact of ART on daily living (low/high), and satisfaction with physical health (satisfied/dissatisfied) [24].

We also assessed several psychosocial factors. HIV-related trauma symptoms were measured using the Impact Event Scale [25]. Based on the distribution, scale scores were dichotomized to subclinical (≤ 8) and symptoms present (9–75). HIV-related stigmatization was measured using a short version of the HIV Stigma Scale [26, 27], including three subscales: internalized, anticipated, and personalized stigma. Scores on the three subscales dichotomized to reflect low stigma (3–7) and high stigma (8–12). The impact of HIV on one’s life was measured by three subscales indicating no impact, HIV negative impact, and HIV positive impact. HIV-related emotional support from partners, family members or friends was dichotomized into high/low. Satisfaction with HIV openness was assessed by a single item having three choices (yes, no, would like to be more open, no, would like to be less open). Impact of the HIV diagnosis on self-image and meaningfulness of life were measured by single items having three choices (weakened, no impact, reinforced).

### Statistical analyses

In total, 1096 eligible individuals responded to the anonymous questionnaire of whom 762 were men, 320 were women, four participants reported ‘‘other’’gender identity, and ten participants did not report their gender. Fourteen participants (4 ‘‘other’’ and 10 missing) were dropped from further analysis, leaving a total of 1082 study participants.

If scores were missing for one or two hopelessness scale items, the missing score was replaced with a participant’s mean scale score. If more than two scale items had missing values, data were excluded from further analyses. The vast majority (89%, n = 967) had complete data on the Beck Hopelessness Scale. After examining responses to the hopelessness scale, we found it to be highly skewed toward lower scores. Therefore, hopelessness was recoded as a binary variable (no feelings of hopelessness (scores 0–3) or feeling hopeless (scores 4–20).

Analyses were conducted separately for women and men in three steps using MPlus version 7.4. To determine which factors were associated with hopelessness, binary logistic regression for sociodemographic characteristics and HIV related clinical and psychosocial measures were calculated. Factors with a statistically significant association with hopelessness were included in a multiple logistic regression model.

Secondly, to establish factors which might indirectly affect hopelessness, binary logistic regressions were conducted between sociodemographic characteristic, HIV related clinical, and psychosocial factors and those factors that were identified as statistically directly associated with hopelessness. Statistically significant variables were then included in multiple logistic regression models. The results of steps one and two provided possible models, one for women and one for men, of hopelessness.

The final analytical step evaluated the fit of the direct and indirect contributors of hopelessness. Separate path models were developed for women and men including only the statistically significant variables from previous analyses using maximum likelihood estimators. Full models were compared to models including all potential predictors using the likelihood difference test, where -2 times the log-likelihood difference is distributed as chi-square [28] and differences in Akaike Information Criteria (AIC) and Bayesian Information Criteria (BIC).

To manage different degrees of missing data, multiple imputation followed by maximum likelihood estimation was being used [29]. Imputation for variables with missing values was conducted using Bayesian analyses [30]. Ten imputed datasets were used in the estimation of all analyses using maximum likelihood estimation. Maximum likelihood parameter estimates for each analysis were averaged over the set of 10 analyses, and standard errors were computed using the average of the standard errors of the analyses and the between analyses parameter estimation.

## Results

Women were younger than men. The average age of women was 43. The average age of men was just shy of 50. Two-thirds of men were native Swedish, while two-thirds of women were born outside Sweden. More than half of men and almost two-thirds of women were in an intimate relationship at the time of data collection. Two thirds of men said they were non-heterosexual, while almost all women said they were heterosexual. Nearly half of men and just over 40% of women had at least some post-high school education. Most men and women were working. Roughly equal percentages of men had been diagnosed with HIV for less than or more than ten years. Differences in time since HIV diagnosis was somewhat greater in women, with the majority have been diagnosed for less than 10 years (Table 1).

**Table 1:** Sociodemographic characteristics of study participants (n = 967)

Variable	Men (n = 699) (%)/mean (SD)	Women (n = 268) (%)/mean (SD)
Age (years)	49.8 (11.7)	42.7 (10.,4)
*Sexual orientation*		
Heterosexual	226 (34.3)	194 (94.6)
Non-heterosexual	433 (65.7)	11 (5.4)
*Country of birth*		
Sweden	477 (69.3)	83 (31.4)
Outside Sweden	211 (30.7)	181 (68.6)
*Current relationship status*		
In an intimate relationship	389 (55.8)	171 (64.8)
Single	308 (44.2)	93 (32.5)
*Years of school*		
≤ 12 years	348 (50.8)	156 (58.9)
≥ 13 years	337 (49.2)	109 (41.1)
*Employment*		
Working	478 (77.0)	178 (70.9)
Non-working	143 (23.0)	73 (29.1)
*Time since HIV diagnosis*		
< 1–10 years	335 (48.7)	142 (54.6)
> 10 years	353 (51.3)	118 (45.4)

Feelings of hopelessness were reported by almost half of study participants. Of these, about half reported feeling mild hopelessness, a quarter moderate hopelessness, and another quarter severe hopelessness. There were no differences in frequency of feeling hopeless or level of hopelessness by gender or sexual orientation.

### Binary and multiple logistic regression analyses predicting hopelessness

The binary logistic regression analyses of factors associated with hopelessness showed dissatisfaction with finances and physical health, and HIV-related emotional support were associated for both women and men (Table 2). For men, but not women, age and being single were associated with hopelessness. The statistically significant binary variables remained significant in the multiple logistic regression models for both genders.

**Table 2:** Binary and multiple logistic regressions predicting hopelessness among male (n = 699) and female (n = 268) study participants

Variable	Binary logistic regression		Multiple logistic regression	
	Male participants OR (95% CI)	Female participants OR (95% CI)	Male participants aOR (95% CI)	Female participants aOR (95% CI)
*Sociodemographic characteristics*				
Age	1.02** (1.00, 1.03)	1.00 (0.96, 1.03)	1.02** (1.01, 1.04)	
Country of birth				
Sweden	1	1		
Outside Sweden	1.34 (0.89, 2.02)	1.38 (0.76, 2.54)		
Relationship status				
In an intimate relationship	1	1	1	
Single	1.48* (1.04, 2.10)	1.69 (0.88, 3.24)	1.55* (1.09, 2.20)	
Sexual orientation				
Heterosexual	1	1		
Non-heterosexual	1.11 (0.72, 1.69)	1.90 (0.39, 9.24)		
Years of school				
≤ 12 years	1.14 (0.79, 1.63)	1.44 (0.82, 2.53)		
≥ 13 years	1	1		
Employment				
Working	1	1		
Non-working	1.52 (0.90, 2.57)	1.33 (0.75, 2.37)		
Financial satisfaction				
Satisfied	1	1	1	1
Dissatisfied	3.33*** (2.15, 5.16)	2.25** (1.25, 4.07)	3.87*** (2.59, 5.80)	2.29** (1.26, 4.14)
*Self-reported HIV clinical factors*				
Time since HIV diagnosis				
≤ 10 years	1	1		
> 10 years	0.97 (0.66, 1.41)	1.24 (0.64, 2.39)		
Experiencing bothersome physical ART side-effects				
Yes	1.43 (0.96, 2.13)	1.72 (0.86, 3.44)		
No	1	1		
Impact of ART on daily living				
High	1.03 (0.71, 1.51)	0.81 (0.44, 1.50)		
Low	1	1		
Physical health satisfaction				
Satisfied	1	1	1	1
Dissatisfied	2.56*** (1.69, 3.88)	3.32*** (1.76, 6.27)	2.70*** (1.80, 4.06)	3.56*** (1.87, 6.81)
*Psychosocial factors*				
HIV-related trauma symptoms				
Subclinical	1	1		
Symptoms present	1.31 (0.89, 1.91)	0.87 (0.46, 1.62)		
Internalized stigmatization				
Low	1	1		
High	0.90 (0.63, 1.31)	0.71 (0.43, 1.18)		
Anticipated stigmatization				
Low	1	1		
High	1.30 (0.88, 1.92)	1.05 (0.55, 2.03)		
Personalized stigmatization				
Low	1	1		
High		0.89 (0.42, 1.87)		
Impact of HIV on one’s life				
No impact	0.92 (0.73, 1.16)	1.05 (0.71, 1.56)		
Negative impact	0.92 (0.74, 1.15)	0.99 (0.69, 1.40)		
Positive impact	0.91 (0.73, 1.15)	0.98 (0.69, 1.40)		
HIV-related emotional social support				
High	1	1	1	1
Low	2.43*** (1.58, 3.74)	2.59** (1.33, 5.04)	2.49*** (1.70, 3.66)	2.56** (1.30, 5.04)
Satisfaction with openness about HIV	0.99 (0.74, 1.33)	1.44 (0.91, 2.29		
Change in sense of self-esteem	0.98 (0.77, 1.24)	0.74 (0.48, 1.13)		
Change in sense of meaningfulness of life	1.00 (0.76, 1.30)	1.41 (0.88, 2.24)		

*OR* odds ratio, *aOR* adjusted odds ratio, *CI* confidence interval, *ART* antiretroviral therapy

*p < 0.05; **p < 0.01; ***p < 0.001

Factors found to be significantly related to hopelessness in women and men were used as dependent variables to assess which factors might be indirectly associated with hopelessness. As shown in Table 3, lower education and being unemployed were associated with dissatisfaction with finances for women. Lower education, unemployed, and anticipated stigmatization were associated with dissatisfaction with physical health. Finally, being single and experiencing bothersome ART side effects were associated with low HIV-related emotional support.

**Table 3:** Predictors of mediating variables of hopelessness, female study participants (n = 268)

	Financial dissatisfaction aOR (95% CI)	Physical health dissatisfaction aOR (95% CI)	Low HIV-related emotional support aOR (95% CI)
*Sociodemographic characteristics*			
Age	0.98 (0.95, 1.00)		
Relationship status			
In an intimate relationship		1	1
Single		1.55 (0.87, 2.76)	3.28*** (1.72, 6.25)
Years of school			
≤ 12 years	1.92* (1.10, 3.35)	2.75** (1.49, 5.10)	
≥ 13 years	1	1	
Employment			
Working	1	1	
Non-working	3.48*** (1.85, 6.52)	2.51** (1.33, 4.71)	
*Self-reported HIV clinical factors*			
Time since HIV diagnosis			
≤ 10 years		1	
≥ 10 years		1.33 (0.76, 2.35)	
Experiencing bothersome physical ART side-effects			
Yes			0.44* (0.22, 0.89)
No			1
*Psychosocial factors*			
Anticipated stigmatization			
Low			1
High			1.89* (1.05, 3.41)

*aOR* adjusted odds ratio, *CI* confidence interval, *ART* antiretroviral therapy

^*^p < 0.05; **p < 0.01; ***p < 0.001

The results for men are shown in Table 4. Being born outside Sweden, unemployed, and time since HIV diagnosis associated with age, being single, dissatisfaction with finances, and dissatisfaction with physical health. Internalized stigmatization and dissatisfaction with openness about HIV were found to be associated with low HIV-related emotional support.

**Table 4:** Predictors of mediating variables of hopelessness, male study participants (n = 699)

	Age aOR (95% CI)	Being single aOR (95% CI)	Financial dissatisfaction aOR (95% CI)	Physical health dissatisfaction aOR (95% CI)	Low HIV-related emotional support aOR (95% CI)
*Sociodemographic characteristics*					
Country of birth					
Sweden	1		1		
Outside Sweden	− 4.76*** (− 6.50, − 3.02)		2.41*** (1.66, 3.61)		
Years of school					
≤ 12 years		0.75 (0.55, 1.03)	1.43 (0.98, 2.07)		
≥ 13 years		1	1		
Employment					
Working	1	1	1	1	
Non-working	5.00*** (2.67, 7.34)	1.77** (1.22, 2.71)	7.21*** (4.54, 11.45)	2.48*** (1.64, 3.76)	
*Self-reported HIV clinical factors*					
Time since HIV diagnosis					
≤ 10 years	1		1		
≥ 10 years	7.58*** (5.96, 9.21)		0.63* (0.43, 0.93)		
Experiencing bothersome physical ART side-effects					
Yes				1.41 (0.94, 2.12)	1.74 (0.98, 2.66)
No				1	1
*Psychosocial factors*					
Internalized stigmatization					
Low					1
High					0.60* (0.42, 0.87)
Anticipated stigmatization					
Low			1	1	
High			0.70 (0.47, 1.04)	0.82 (0.58, 1.15)	
Personalized stigmatization					
Low			1		
High			1.42 (0.92, 2.18)		
Satisfaction with openness about HIV					0.72* (0.55, 0.94)

*aOR* adjusted odds ratio, *CI* confidence interval, *ART* antiretroviral therapy

*p < 0.05; **p < 0.01; ***p < 0.001

### Path models

**Fig. 1:**
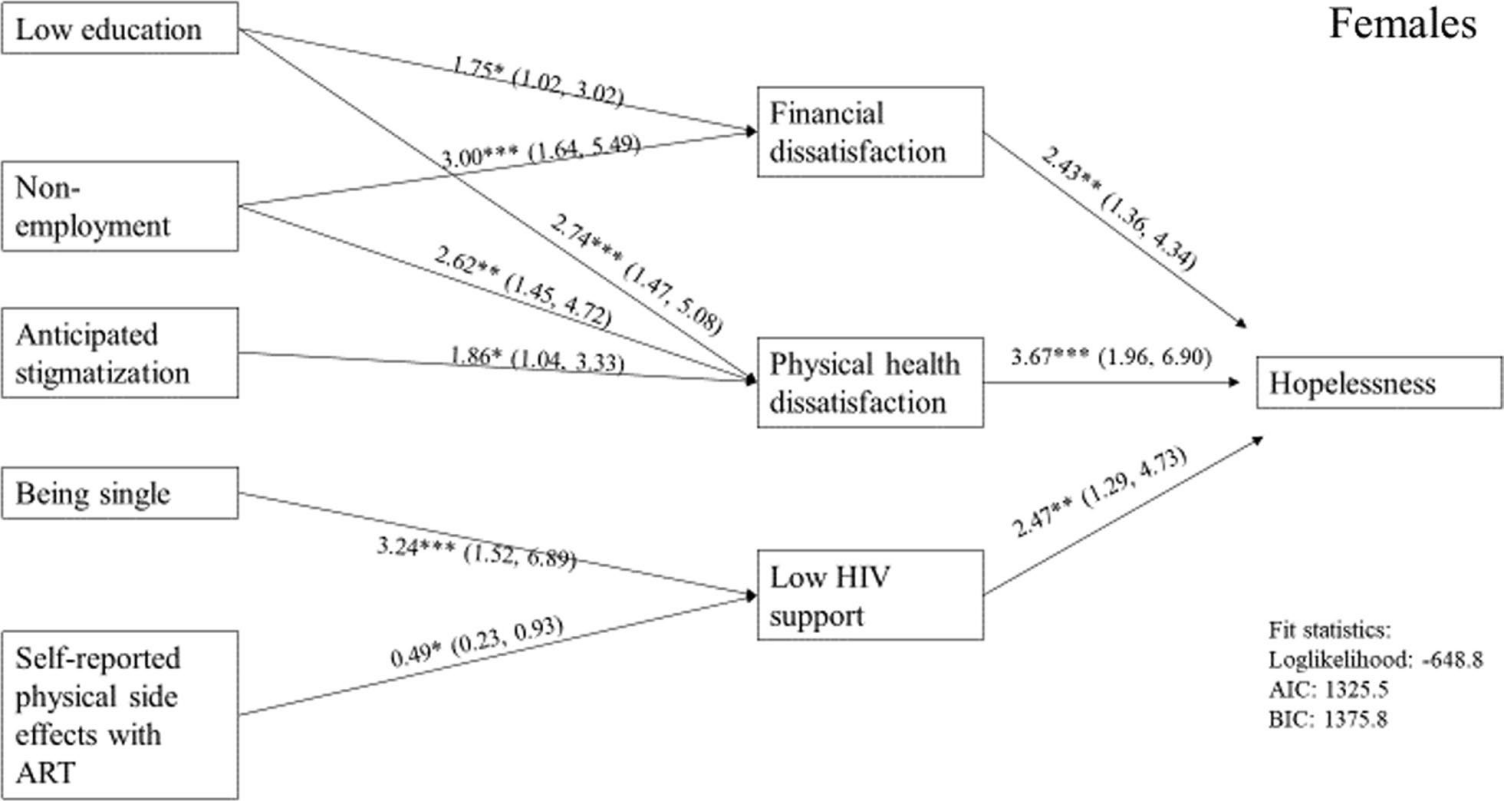
Path diagram, female participants (n = 268)

**Fig. 2:**
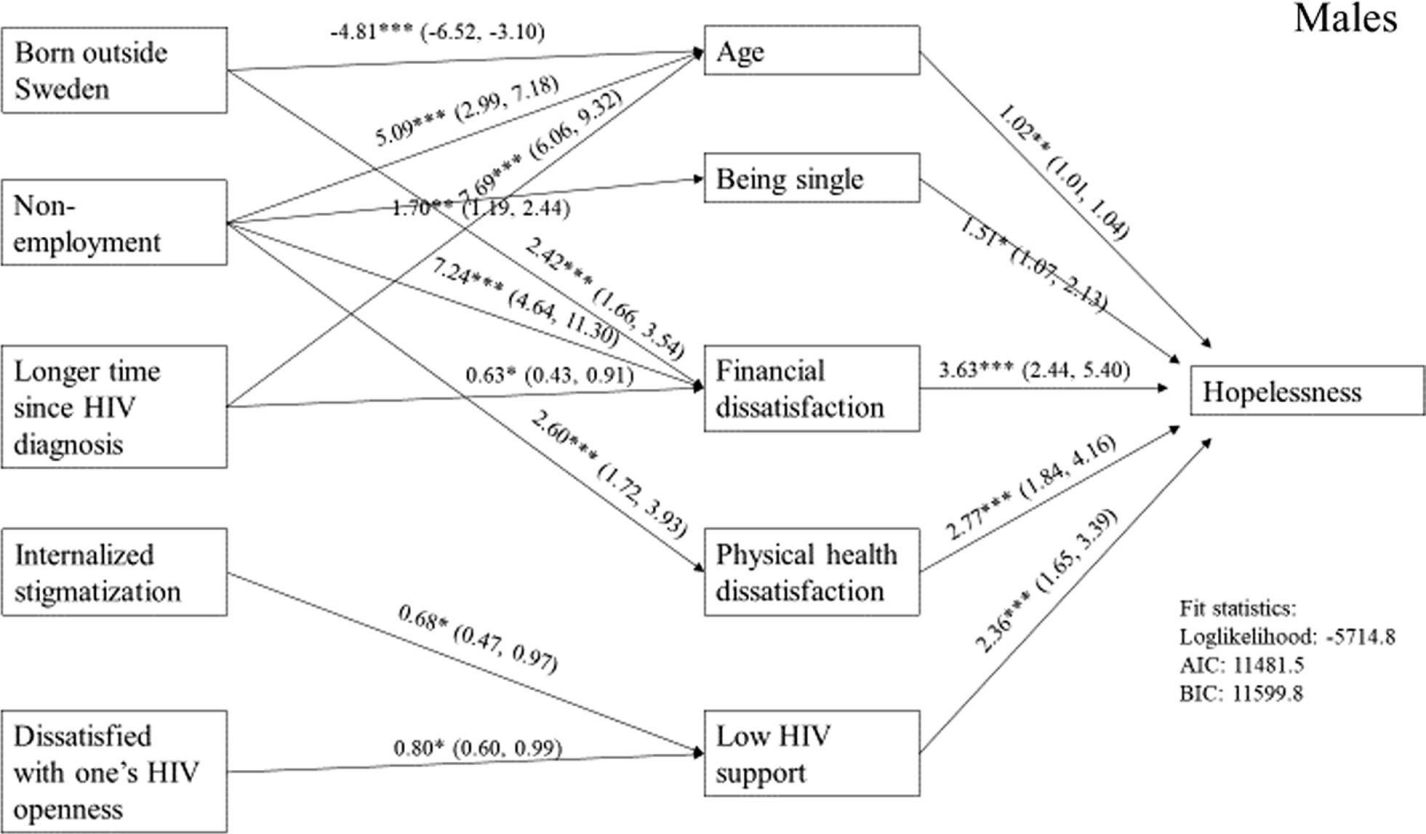
Path diagram, male participants (n = 699)

The binary and multiple analyses suggested hopelessness path models that could be tested. The path models for women and men are show in Figs. 1 and 2. Both models showed good fit. All factors found to have a significant association with hopelessness or as an indirect factor remained significant in path models.

Results showed dissatisfaction with physical health had the strongest direct path to hopelessness in women. Dissatisfaction with finances and low HIV-related emotional support were also found to be significant and equally strong. In men, the same paths were found to be significant, but with different strengths than in the women’s model. The strongest path was dissatisfaction with finances, followed by dissatisfaction with physical health and low HIV-related emotional support. Being single was the fourth strongest path, followed by age.

Indirect paths to hopelessness were quite different for women and men. Among women, paths from low education and unemployed were found to dissatisfaction with finances and physical health. The only significant indirect path from anticipated stigmatization was found to dissatisfaction with physical health. The path from being single to low HIV-related emotional support was found to be strong, while the path from bothersome ART sides effects to low HIV-related emotional support was significant but weak.

For men, the model showed several paths from being unemployed. The strongest path was from unemployment to dissatisfaction with finances. Other paths from unemployment were to age, being single, and dissatisfaction with physical health. Two paths were significant from born outside Sweden. The strongest was to age followed by dissatisfaction with finances. Two paths were also found to be significant from time since HIV diagnosis. The path from time since HIV diagnosis to age was found to be very strong. The path from time since HIV diagnosis to dissatisfaction with finances was weak. Only one path was found to be significant from internalized stigmatization and dissatisfaction with openness about HIV. Both paths ran from these variables to low HIV-related emotional support.

## Discussion

The aim of this study was to assess prevalence and levels of hopelessness and its underlying structure in a sample of women and men living with HIV in Sweden. We found feelings of moderate to severe hopelessness in about half of both women and men, which is much higher than in studies involving general populations [10, 31]. This suggests feelings of hopeless are relatively common in people living with HIV, and may indicate, as some have suggested, an elevated risk of suicidal ideation [2, 32, 33]. As at least one other study, we found no differences in prevalence or level of hopelessness between women and men [2].

Nevertheless, we found different underlying structures of hopelessness in women and men, although the path models for each did share common factors contributing directly to hopelessness, that is dissatisfaction with one’s physical health, dissatisfaction with one’s finances, and low HIV-related emotional support.

Being dissatisfied with one’s physical health was a strong predictor of hopelessness. This suggests predictors of feelings of hopelessness are related to the effect HIV is having on the body and demonstrates a strong connection between the physical and psychological [16]. Such a relationship is also found in the area of other chronic conditions such as cardiovascular diseases, cancer, and diabetes [34].

Dissatisfaction with one’s physical health was the strongest predictor of hopelessness in women, but not men, suggesting women may feel negative health outcomes somewhat more strongly.

Dissatisfaction with finances suggests financial hardship is strongly associated with feelings of hopelessness. Whether this is related to feelings of lower socio-economic status or difficulties to independently provide for one’s self is unclear [14, 35]. For men, dissatisfaction with finances presented the strongest pathway and, as suggested by the model, is strongly related to unemployment.

Low HIV-related emotional support was almost an equally strong predictor of hopelessness for both men and women. It may be a consequence of concealing one’s HIV status out of fear of stigmatization and rejection or the experience of other’s withdrawal upon HIV disclosure. This finding strongly indicates having an emotional social support system is important for maintaining psychological wellbeing in people living with HIV [8, 11, 15]. The significance of emotional social support is a well-known fact also in other chronic [36] and acute [37] health condition as well as in the general population [38].

Being single and increasing age predicted hopelessness in men, but not women. The paths from these two predictors to hopelessness were not strong, especially the path from age. The association between being single and feelings of hopeless in men has been observed by at least one other study [14]. We do not know whether being single was HIV-related or of a circumstantial or intentional character. There may be a longing for an intimate relationship, but HIV puts up barriers to fulfill this wish [17]. As intimate relationship functions as a protective shield in stressful times, being single may trigger feelings of loneliness. Research acknowledges loneliness as a central aspect of life among people living with HIV [39], but also for people with cardiovascular, cerebrovascular, and other chronic diseases [40]. The association between loneliness and depression is well documented [39–41].

Increasing age has a number of possible explanations. Adverse psychological effects of HIV as well as potential chronic health problems may be common among older people with HIV [42]. Increasing age may also be indicative of increased feelings of vulnerability to social isolation, existential issues such as meaning of life [43], existential isolation [44], and feelings associated with dying and death.

Women presented a comparatively simpler underlying structure of hopelessness than men, in that fewer paths were found to significantly influence feelings of hopeless through direct factors. Men had a more complex structure, but this was due largely to employment status. For both women and men, the strongest indirect path was from unemployment to dissatisfaction with finances, strongly suggesting unemployment indirectly has a significant impact on psychological wellbeing [35, 45–47]. Whether unemployment is to lack of a job, long-term sick leave, disability pension, or pension is unclear from the data. Even so, unemployment is likely indicative of economic strain, discontent with circumstances, and possibly feeling decreased social status associated with not being a “working person” [48]. It is also possible unemployment may associate with physical limitations due to HIV [47].

While HIV stigmatization was indirectly associated with hopelessness for both women and men, type of stigmatization and its indirect paths differed by gender. Among women, anticipated stigmatization, which may be perceived as a stressor, had an indirect effect on hopelessness through dissatisfaction with physical health. Our data supports other studies that anticipated stigmatization undermines the physical and mental well-being [49, 50]. For men, internalized HIV stigmatization indirectly affected hopelessness, similar to another study [12]. Characteristics of internalized HIV stigmatization are among others self-loathing and feelings of being inferior to others. These may be strong barriers to HIV disclosure reflected in low HIV-related emotional support, leading to social isolation and loneliness. Other than having unemployment and HIV stigmatization in common, the factors indirectly associated with hopelessness were different in men and women.

For women, there were three indirect paths to hopelessness: being single, lower education, and physical side effects from ART. The strongest indirect path was from being single to low HIV-related emotional support, which is somewhat difficult to understand, as there may be several possible explanations. But, irrespective of its underlying cause, low emotional support may lead to feelings of being excluded and increased vulnerability to social isolation, loneliness and increased psychological distress. Lower level of education had a strong indirect path to physical health dissatisfaction, suggesting lower education indirectly has a significant negative impact on psychological wellbeing [51]. There was also an indirect path from lower level of education to financial dissatisfaction. Less education may be a surrogate measure for lower socio-economic status [35, 51]. Self-reported physical side effects from ART had through low HIV-related emotional support an indirect, but weak effect on hopelessness. Other studies have observed direct association between physical side effects and hopelessness [13, 16].

For men, three factors were indirectly associated with feelings of hopeless: born outside Sweden, time since HIV diagnosis, and dissatisfaction with one’s openness about HIV. There were two indirect paths from born outside Sweden to hopelessness. The strongest was to age, suggesting foreign born men may have been younger than Swedish born men living with HIV. This is supported by the Swedish HIV statistics [52]. The other indirect path from born outside Sweden was to dissatisfaction with finances. Migrants in Sweden are often economically more vulnerable than Swedish-born citizens [53]. Their financial hardship may be indicative of the combination of migration and HIV status, suggesting being a migrant indirectly increases psychological distress [49, 53, 54]. In contrast to Stanley et al.’s study [14], time with diagnosed HIV infection had a strong indirect effect on hopelessness through age. Older men were likely to have been diagnosed with HIV infection for more than 10 years, signifying they may have ongoing health problems or disability related to HIV infection [55]. The indirect path from time since HIV diagnosis to financial dissatisfaction was weak, but may reflect increased time living with HIV has had its toll on the financial situation. Dissatisfaction with one’s openness about HIV had a weak association through low HIV-related emotional support to feelings of hopelessness.

### Strengths and limitations

The strength of this study is its nationwide, multicultural sample, including almost a quarter of these who could have attended the 17 HIV units in Sweden at the time of data collection. Nevertheless, given the cross-sectional design, cause and effect relationships are not possible. Furthermore, underlying structures of hopelessness may change over time given that feelings of hopelessness are dynamic. Another limitation is response bias. We don’t know about the people who didn’t enroll, and why not. Or what proportion didn’t visit a clinic in that time frame. This study also contained a smaller proportion of women (28% vs. 38% overall) and a higher proportion of Swedish born participants (59% vs. 36% overall) than the national demographic of people living with HIV. These differences may decrease the generalizability of the findings to the HIV population in Sweden. Considering the average age of the participants were higher in men (43 for women and approximately 50 for men) the question remains if a younger study population would have yielded different results. Nevertheless, the average age of women and men in our sample corresponds with the age distribution of the larger population of people living with HIV in Sweden at the time of data collection. Because the sample was drawn only from persons living with HIV in Sweden, results are not generalizable to populations living elsewhere. As the data were self-reported, they may include the social desirability bias, which may have decreased given the anonymous questionnaire. We did not measure depression and it is possible that it would co-vary with feelings of hopelessness. Further studies are needed to assess their associations and underlying structures.

## Conclusions

Our study adds to the limited research on hopelessness and its correlates among people living with HIV by examining direct and indirect correlates of hopelessness by gender. Despite successful antiretroviral treatment, the study shows that people living with HIV in Sweden experience high levels of hopelessness. Our findings indicate few gender differences in direct contributors to hopelessness, but different path models of indirect factors. In contrast to research on corelates of depression, we did not find any significant associations between gender, younger age, post-traumatic stress, HIV stress and hopelessness [56]. This suggests feelings of hopelessness deserve to be studied independently of depression and more studies are warranted.

Our study clearly signifies that HIV is not a normal, chronic disease like any other, but a psychosocial condition and not just a medical one [57]. Our findings are important to HIV clinicians in identifying those at risk of hopelessness from a gender perspective in order to reduce preventable psychological distress among people living with HIV.

## Data Availability

The data underlying the findings in our study are not publicly available because the original approval by the Regional Ethical Board, Stockholm, Sweden. (DNR 2013/1552-31/4) and the informed consent from the subjects participating in the study did not include such a direct, free access.
